# A weight optimization-based transfer learning approach for plant disease detection of New Zealand vegetables

**DOI:** 10.3389/fpls.2022.1008079

**Published:** 2022-10-25

**Authors:** Muhammad Hammad Saleem, Johan Potgieter, Khalid Mahmood Arif

**Affiliations:** ^1^ Department of Mechanical and Electrical Engineering, School of Food and Advanced Technology, Massey University, Auckland, New Zealand; ^2^ Massey AgriFood Digital Lab, Massey University, Palmerston North, New Zealand

**Keywords:** convolutional neural networks, deep learning, transfer learning, optimization algorithms, cross-validation, plant disease detection

## Abstract

Deep learning (DL) is an effective approach to identifying plant diseases. Among several DL-based techniques, transfer learning (TL) produces significant results in terms of improved accuracy. However, the usefulness of TL has not yet been explored using weights optimized from agricultural datasets. Furthermore, the detection of plant diseases in different organs of various vegetables has not yet been performed using a trained/optimized DL model. Moreover, the presence/detection of multiple diseases in vegetable organs has not yet been investigated. To address these research gaps, a new dataset named NZDLPlantDisease-v2 has been collected for New Zealand vegetables. The dataset includes 28 healthy and defective organs of beans, broccoli, cabbage, cauliflower, kumara, peas, potato, and tomato. This paper presents a transfer learning method that optimizes weights obtained through agricultural datasets for better outcomes in plant disease identification. First, several DL architectures are compared to obtain the best-suited model, and then, data augmentation techniques are applied. The Faster Region-based Convolutional Neural Network (RCNN) Inception ResNet-v2 attained the highest mean average precision (mAP) compared to the other DL models including different versions of Faster RCNN, Single-Shot Multibox Detector (SSD), Region-based Fully Convolutional Networks (RFCN), RetinaNet, and EfficientDet. Next, weight optimization is performed on datasets including PlantVillage, NZDLPlantDisease-v1, and DeepWeeds using image resizers, interpolators, initializers, batch normalization, and DL optimizers. Updated/optimized weights are then used to retrain the Faster RCNN Inception ResNet-v2 model on the proposed dataset. Finally, the results are compared with the model trained/optimized using a large dataset, such as Common Objects in Context (COCO). The final mAP improves by 9.25% and is found to be 91.33%. Moreover, the robustness of the methodology is demonstrated by testing the final model on an external dataset and using the stratified k-fold cross-validation method.

## Introduction

According to the guidelines of the World Health Organization (WHO) and the Food and Agriculture Organization (FAO), fruit and vegetable consumption should be greater than 400 g/day to reduce the risk of heart disease, high blood pressure, and stroke (Who and Consultation, 2003). Therefore, adequate food supply must be achieved for human well-being. In this regard, food security is essential to ensure food demand. Plant diseases are a substantial threat to food security (Gui et al., 2021) and affect crop productivity (Liu and Wang, 2021). They also influence the quality of agricultural products and are a source of economic loss.

Horticultural products are among the main contributors to New Zealand (NZ) economy. According to a recent report by Plant and Food Research, the export value of fresh and processed vegetables was estimated to be NZ$724.5m in 2020 (Aitken and Warrington, 2020). Among the most prominent vegetables, onions, peas, potatoes, squash, sweetcorn, and beans were exported in amounts of NZ$147.6m, NZ$115.4m, NZ$106.9m, NZ$79.2m, NZ$47.6m, and NZ$42.0m, respectively. The highest expenditures for imported vegetables were observed for preserved tomatoes and frozen potatoes at around NZ$35.8m and NZ$34.9m, respectively. A total of NZ$1.29b was recorded for the vegetables consumed for domestic use, such as potatoes, tomatoes, and brassicas (broccoli, cabbage, and cauliflower). Furthermore, the largest horticultural land (among the vegetables) was used for potatoes around 10,417 ha, 6,530 ha for squash, 5,296 ha for onion, and 4,890 ha for peas and beans (Aitken and Warrington, 2020). These statistics highlight the importance of NZ vegetables to the country’s income. Therefore, the challenges faced by the horticultural industry should be addressed to increase the export value of horticultural products further.

Plant disease detection is an important task in the application of control treatments to the affected plants. However, the timely and precise identification of plant diseases is challenging. This is due to the similarity in the occurrence of the disease in different plant species. Therefore, the traditional methods for recognizing plant diseases have been replaced by methods based on machine learning (ML). Among these techniques, deep learning (DL) has gained considerable attention from the scientific community in recent years, and numerous DL methods, architectures, and approaches have been proposed. However, current literature leaves a significant margin for further investigating the strength of DL-based plant disease identification in various ways.

The research done so far for plant disease detection has covered various aspects of deep learning, such as DL-based visualization techniques to identify the spots of plant disease (Brahimi et al., 2018; Brahimi et al., 2019) and the application of the latest data augmentation techniques (Bi and Hu, 2020; Abbas et al., 2021). Furthermore, some studies have focused on modifications in the hidden layers of neural networks (Kamal et al., 2019; Liang et al., 2019; Liu and Wang, 2020), improvement in the feature fusion module (Bao et al., 2022), and the addition of attention modules (Wang et al., 2021a; Bao et al., 2022). Moreover, few articles have addressed the real-time assessment of the DL model for the recognition of plant disease (Xie et al., 2020; Wang et al., 2021b) and the evaluation of the usefulness of deep learning optimizers (Saleem et al., 2020).

Transfer learning (TL) is a successful technique for improving the performance of DL models. In TL, the knowledge gained from the pre-trained weights of a large dataset is utilized to extract the specific features of the dataset that contains new classes. A few studies have presented the significance of TL in plant disease identification. For example, the effectiveness of the fine-tuning technique was shown for plant disease classification (Brahimi et al., 2017; Too et al., 2019; Hassan et al., 2021), using pre-trained weights in a large dataset called ImageNet. Another study evaluated TL on synthetic images (generated by a generative adversarial network) and real images (Abbas et al., 2021). Although these articles presented the importance of fine-tuning, the selected dataset had a plain background/controlled environment. Only one study has performed a plant disease identification task in a real agricultural environment using a transfer learning approach (Chen et al., 2020a). Another study considered field conditions for the detection of diseases in maize plants using three models inspired by Inception-v3 (Haque et al., 2022). The performance of the proposed model was better than that of the pre-trained DL models. However, the DL architecture required a longer computation time. A study presented eggplant disease detection using pre-trained weights of the visual geometry group (VGG) model and created a new dataset with a combination of controlled/laboratory and uncontrolled/real agricultural environments (Krishnaswamy Rangarajan and Purushothaman, 2020). Another study presented TL by combining the DenseNet model and Inception module for rice plant diseases under actual conditions (Chen et al., 2020c). A two-phase approach to applying TL was presented by (Chen et al., 2020b). The first phase was dedicated to training from scratch for the new layers and using pre-trained weights for the rest of the layers, whereas the second phase consisted of retraining the model on the selected dataset using weights obtained in the previous stage. Although a comprehensive analysis was provided, the comparison of the proposed method with other modified versions of the DL architecture could further strengthen the quality of the work. In a similar style, a recent study proposed a transfer-learning-based deep feature descriptor (Fan et al., 2022). The authors demonstrated the effectiveness of the approach on different datasets and showed the novelty of this work. Another study presented the significance of TL in tomato plant disease identification (Thangaraj et al., 2021). In this work, a few of the models were included for comparative analysis with the proposed model. A study evaluated the TL by splitting the data into the source and target domains (Argüeso et al., 2020). This study provided a new approach to exploring TL-based techniques. However, it should be validated in other datasets related to plant diseases. Another study evaluated TL based on domain splitting and analyzed semi-supervised iterations and few-shot parameters (Rukundo, 2021). This research presented validation using three domain splits of the same dataset, whereas a more comprehensive analysis could be included, such as considering different datasets with diverse environments. An article presented a multitask TL approach to identify rice and wheat plant diseases at the same time, compared to other techniques including a single task, a reuse model, and some DL models (Jiang et al., 2021). Comparison of TL using pre-trained weights on a large dataset consisting of general objects and a dataset related to plant recognition was presented by (Lee et al., 2020). This proved to be an innovative method to demonstrate the importance of transfer learning. It was also observed that a more in-depth analysis could be performed using training profiles.

The literature references presented above have considerable scope for exploring transfer learning methods to improve plant disease detection. The use of image resizers, weight initialization, and DL optimization methods have not been explored for transfer learning purposes. In addition, weight optimization has also not been performed using the datasets related to the same and different agricultural operations. Moreover, most articles have addressed the recognition of plant diseases in a single vegetable under real agricultural conditions. However, the effectiveness of deep transfer learning has not yet been evaluated for different vegetables using the same optimized DL model.

This article addresses several concerns regarding deep transfer-learning-based plant disease detection. The first research question is how well can deep learning detect plant diseases in various organs of vegetables? Can transfer learning be applied for the simultaneous identification of multiple diseases in various vegetable organs at the same time? How do the different methods related to image processing and weight optimization affect the performance of deep transfer learning? Can the weights obtained from the agricultural datasets contribute to improving the performance of the DL model? Connected to the previous question, what are the circumstances to obtain improvement in terms of the dataset conditions? Finally, can the highlighted questions be validated by testing on an external dataset with the same classes and retaining a high mean average precision?

To answer these questions, this article presents a new dataset (the second version in a series of datasets containing plant diseases in various NZ horticultural crops), named NZDLPlantDisease-v2. The generated dataset contains diseases in eight important NZ vegetables, including potatoes, tomatoes, beans, peas, kumara, and brassicas (broccoli, cabbage, and cauliflower). The dataset also contains diseases in different plant organs (leaves, stems, and vegetables), along with multiple diseases in plant organs at a time. Moreover, this study proposes transfer-learning-based plant disease identification. For this purpose, the weights of the best-obtained DL architecture are optimized using a framework consisting of various techniques. These methods include the evaluation of image resizers, interpolators, weight initializers, batch normalization, and DL optimizers. This framework has been derived from our recently presented work on weed detection (Saleem et al., 2022b). Furthermore, optimized weights are obtained using agricultural (similar and/or other applications) datasets. In this regard, three datasets are selected, two of which are related to plant diseases in both laboratory and real field environments, and the last is a weed-related dataset. In addition, weight optimization is performed on a large dataset called common objects in context (COCO). The mean average precisions of the weights obtained using the agricultural and large datasets are compared. Finally, the usefulness of the research is demonstrated by testing on an external dataset and using the stratified k-fold cross-validation method. Hence, this article provides new insights into DL-based plant disease recognition rather than overly explored open-source datasets such as PlantVillage.

The main contributions of this research are as follows: (1) a new dataset is presented consisting of eight prominent vegetables of the NZ and it has been made publicly available to the scientific community; (2) several conditions and problems of the real horticultural field have also been considered, including the presence of disease in various organs of the vegetable plants, different environmental conditions, and the occurrence of multiple diseases in a vegetable plant organ at a time; (3) a new way of exploiting various techniques has been provided by the integration of weight optimization and transfer learning methods; (4) a high mean average precision (mAP) has been achieved by comparing with the results obtained through pre-trained weights of the COCO dataset; an average precision (AP) of >80% for each class has also been achieved; (5) the robustness of the presented work has been validated by two methods: testing on an externally generated dataset in different agricultural conditions and using the stratified five-fold cross-validation method.

## Materials and methods

### A transfer learning-based approach

The proposed approach is based on transfer learning for the extraction of features of plant diseases. The weights of the best-obtained DL model are updated using the optimized weights from the agricultural dataset. Performance optimization techniques are applied as a bridge to update/optimize the weights of the DL model. The proposed methodology is divided into two phases. Each phase comprises two steps, and the final validation/effectiveness of the approach is presented using two different methods.

The first step in the initial phase of the research was the generation of the dataset called NZDLPlantDisease-v2. The proposed dataset contains eight important vegetables from New Zealand, including 28 healthy and diseased classes. This dataset contains various practical problems in the horticultural field. The second step was a comparative evaluation of various DL meta-architectures to obtain the best DL model in terms of training, validation losses, and mean average precision (mAP). This step of the proposed methodology uses the TL technique by training the models using pre-trained weights on the COCO dataset to extract the basic features. The performance of the best-obtained model was enhanced by applying category-wise data augmentation techniques, including color change (brightness, contrast, and sharpness), translational and rotational changes, and the addition of noise to the color change.

The second phase began with the selection of agricultural datasets. In this regard, two datasets of healthy and defective plant organs and a dataset of weed images were selected. One of the selected plant disease datasets has a plain background, whereas the other has a natural/complex agricultural background. This was done to analyze the effects of the background elements for transfer learning purposes. The idea was to extract the relevant features of plant disease by leveraging the knowledge gained by the DL model from agricultural datasets. In this regard, the next step was weight optimization utilizing our recently presented weed-detection pipeline on these three agricultural datasets.The pipeline/framework consists of five steps (Saleem et al., 2022b): studying the effects of image resizing techniques such as fixed-shape and aspect ratio resizers; combining image interpolators such as bilinear, bicubic, area, and nearest neighbor; application of weight initializers including truncated normal, scaling variance, and random normal initializers; understanding the effects of the absence/presence of batch normalization; and use of DL optimizers such as stochastic gradient descent (SGD) with momentum, root mean square propagation (RMSProp), and adaptive moment estimation (Adam). In this step, the weights of the DL model are updated. Subsequently, the optimized weights of the best-obtained DL model were used to transfer the knowledge for the NZDLPlantdisease-v2 dataset. The mAP attained in this step was compared with that obtained in the first phase of the study. To validate the results, the same optimization pipeline was applied directly to the NZDLPlantDisease-v2 dataset using pre-trained weights on the COCO dataset. A higher AP of the healthy/disease classes established the proposed hypothesis that the best DL architecture is initially trained with a large general-purpose dataset, optimized with image resizing techniques and weight optimization algorithms through agricultural datasets, and retrained through updated weights on the proposed dataset attained better identification of plant diseases.

**Figure 1:**
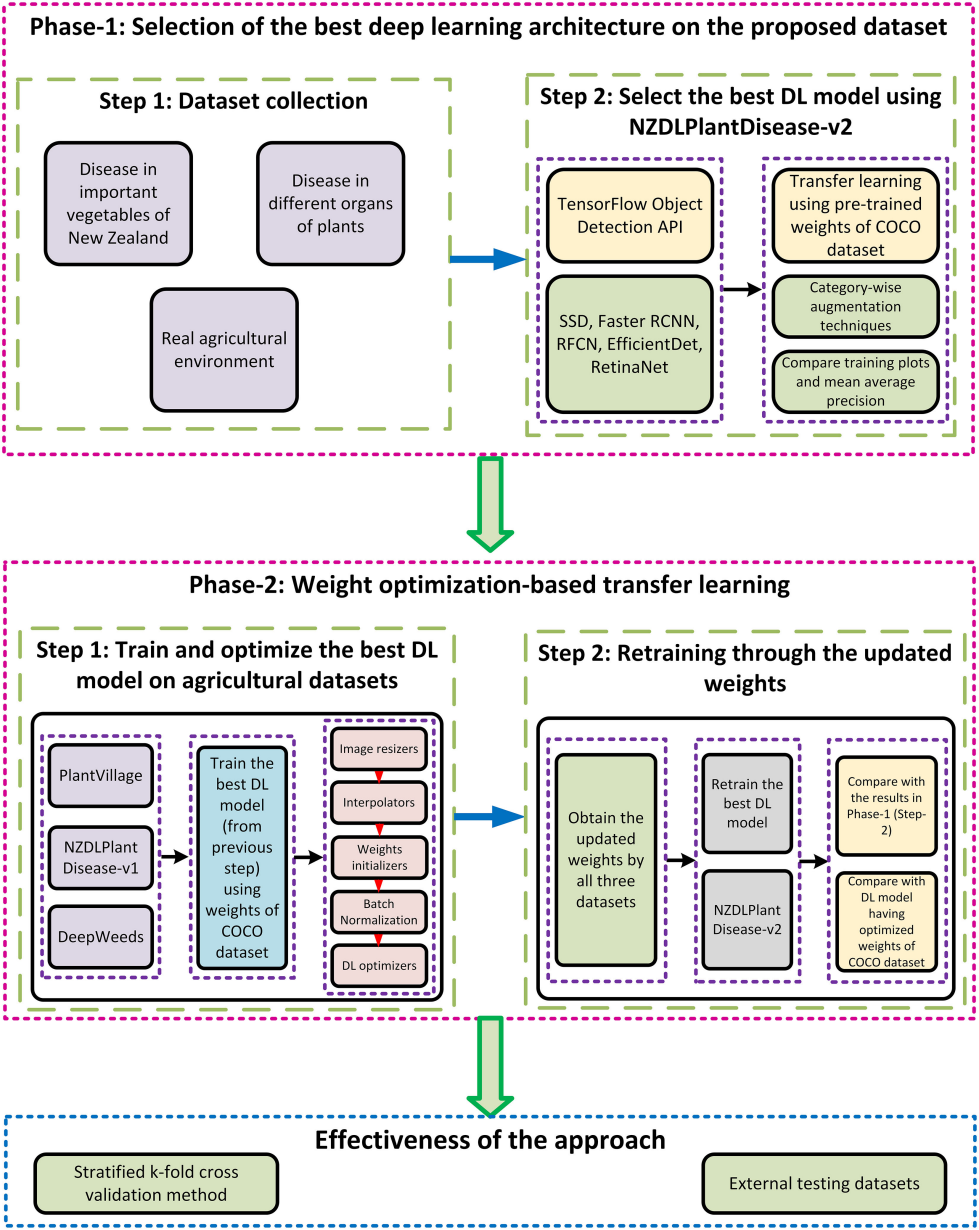
Overall workflow of the proposed methodology.

Finally, the effectiveness of the research was demonstrated by testing the optimized model with an externally generated dataset and using a stratified five-fold cross-validation technique. The research methodology is illustrated in **Figure 1**.

### Proposed dataset

This research presents a new dataset called NZDLPlantDisease-v2, which contains diseases in eight prominent NZ vegetables, including tomato, potato, peas, beans, cabbage, cauliflower, broccoli, and kumara. The Samsung Galaxy S10 Plus smartphone with the following specifications was used to take the dataset images: three cameras with 12 MP f/1.5-2.4 (wide), 12 MP f/2.4 (telephoto), and 16 MP, f/2.2 (ultrawide). Dataset images were collected from various local horticultural fields in Auckland and Palmerston North, New Zealand. The working distance of the images is 200-300 mm. The data collection period was from December 2020 to May 2021.

**Figure 2:**
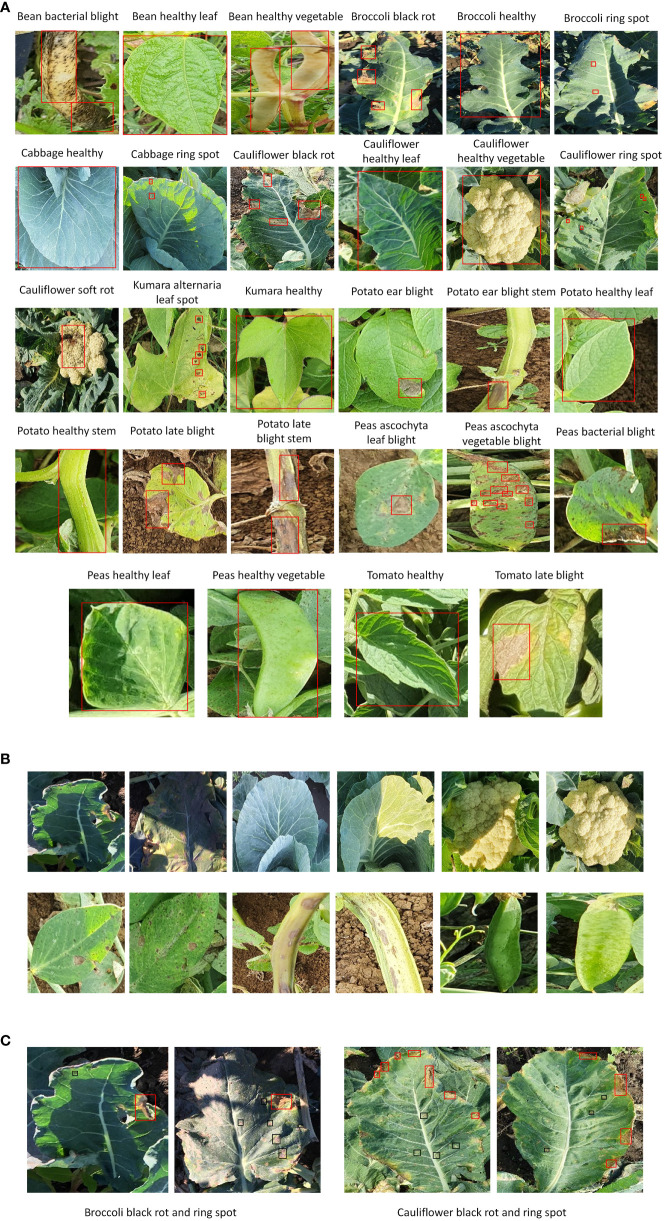
Samples of NZDLPlantDisease-v2 dataset. **(A)** Annotated example of each class. **(B)** Examples of healthy and defective organs of vegetables in different environmental conditions. **(C)** Examples of multiple diseases in plant leaves.

The dataset was divided into three sub-datasets: training (70%), validation (20%), and testing (10%). An open-source tool LabelImg was used to annotate the dataset images. According to the requirements of the TensorFlow object detection API, the XML files were converted into CSV files and then transformed into TFrecords. A sample of each annotated class is shown in **Figure 2A**. Various real agricultural conditions, such as the absence/presence of shadows, different lighting conditions, and sudden weather changes, were considered, as shown in **Figure 2B**. Moreover, other practical conditions have been considered by imaging plant organs with multiple diseases. For example, black rot and ring spot disease in broccoli and cauliflower leaves were present simultaneously (**Figure 2C**). However, some crops contain the disease only in their leaves. The details of the proposed dataset are presented in **Table 1**.

**Table 1:** Summary of NZDLPlantDisease-v2 dataset.

Crops	Plant Organs	Pathogens	NZDLPlantDisease-v2 classes with number of images	Simplified annotation labels
Bean	Leaf	–	Healthy (52)	B_healthy_l
	Vegetable	Bacteria	Bacterial blight - Xanthomonas axonopodis pv. phaseoli (93)	B_bac_blight_v
		–	Healthy (55)	B_healthy_v
Broccoli	Leaf	Bacteria	Black rot - Xanthomonas campestris pv. campestris (53)	Br_blk_rot
		Fungi	Ring spot - Mycosphaerella brassicicola (83)	Br_r_spot
		–	Healthy (74)	Br_healthy
Cabbage	Leaf	Fungi	Ring spot - Mycosphaerella brassicicola (192)	C_r_spot
		–	Healthy (173)	C_healthy
Cauliflower	Leaf	Bacteria	Black rot - Xanthomonas campestris pv. campestris (144)	Cf_blk_rot
		Fungi	Ring spot - Mycosphaerella brassicicola (48)	Cf_r_spot
		–	Healthy (68)	Cf_healthy_l
	Vegetable	Bacteria	Soft rot - Erwinia carotovora subsp. carotovora (36)	Cf_s_rot
		–	Healthy (96)	Cf_healthy_v
Kumara	Leaf	Fungi	Alternaria leaf spot - fungus Alternaria spp. (50)	K_alt_lf_spot
		–	Healthy (103)	K_healthy_l
Potato	Leaf	Fungi	Ear blight - Alternaria solani (119)	Po_ear_blight
		Oomycete	Late blight - Phytophthora infestans (57)	Po_lt_blight
		–	Healthy (199)	Po_healthy_l
	Stem	Fungi	Ear blight - Alternaria solani (144)	Po_ear_blight_s
		Oomycete	Late blight - Phytophthora infestans (89)	Po_lt_blight_s
		–	Healthy (107)	Po_healthy_s
Peas	Leaf	Fungi	Ascochyta blight - Mycosphaerella pinodes (224)	Ps_asc_blight
		Bacteria	Bacterial blight - Pseudomonas syringae pv. pisi (68)	Ps_bac_blight
		–	Healthy (115)	Ps_healthy_l
	Vegetable	Fungi	Ascochyta blight - Mycosphaerella pinodes (143)	Ps_asc_blight_v
		–	Healthy (101)	Ps_healthy_v
Tomato	Leaf	Oomycete	Late blight - Phytophthora infestans (133)	T_lt_blight
		–	Healthy (220)	T_healthy_l

### Data augmentation

**Figure 3:**
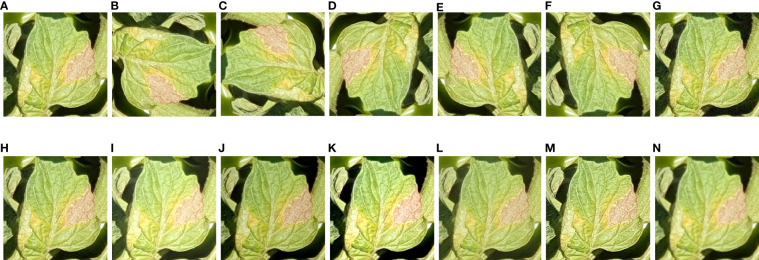
Examples of tomato late blight with all augmentation techniques (including original image). **(A)** Original. **(B)** 90°. **(C)** -90°. **(D)** 180°. **(E)** Horizontal. **(F)** Vertical. **(G)** Gaussian noise, **(H)** Laplacian noise **(I)** High brightness. **(J)** Low brightness. **(K)** high contrast. **(L)** low contrast. **(M)** high sharpness. **(N)** low sharpness.

The size of the dataset was initially increased by cropping a group of healthy/disease classes containing more than one object of interest. Several data augmentation methods were applied, such as a 30% change (increase or decrease) in brightness, sharpness, and contrast (Wang et al., 2021a). In addition, the effects of noise, including Gaussian and Laplacian noise, were evaluated. For this purpose, open-source software named XnViewMP was used. The maximum intensities were 10.0, and 50.0, out of which random intensities of 2.0 and 10.0 were respectively selected. Furthermore, more diversity in the dataset was obtained by rotational/translational changes, such as 90°, -90°, 180°, horizontal, and vertical changes. An example of augmented images for the tomato late blight class is shown in **Figure 3**.

For a comprehensive analysis, the data enhancement methods were arranged into five groups. The first contains only original (OO) images, then a combination of original with translational/rotational changes (OT), original with color variation (OC) (increase/decrease in brightness, contrast, and sharpness), original with noise and variation in color change simultaneously (OCN), and finally a combination of all the methods (OTCN).

### Agricultural datasets

**Figure 4:**
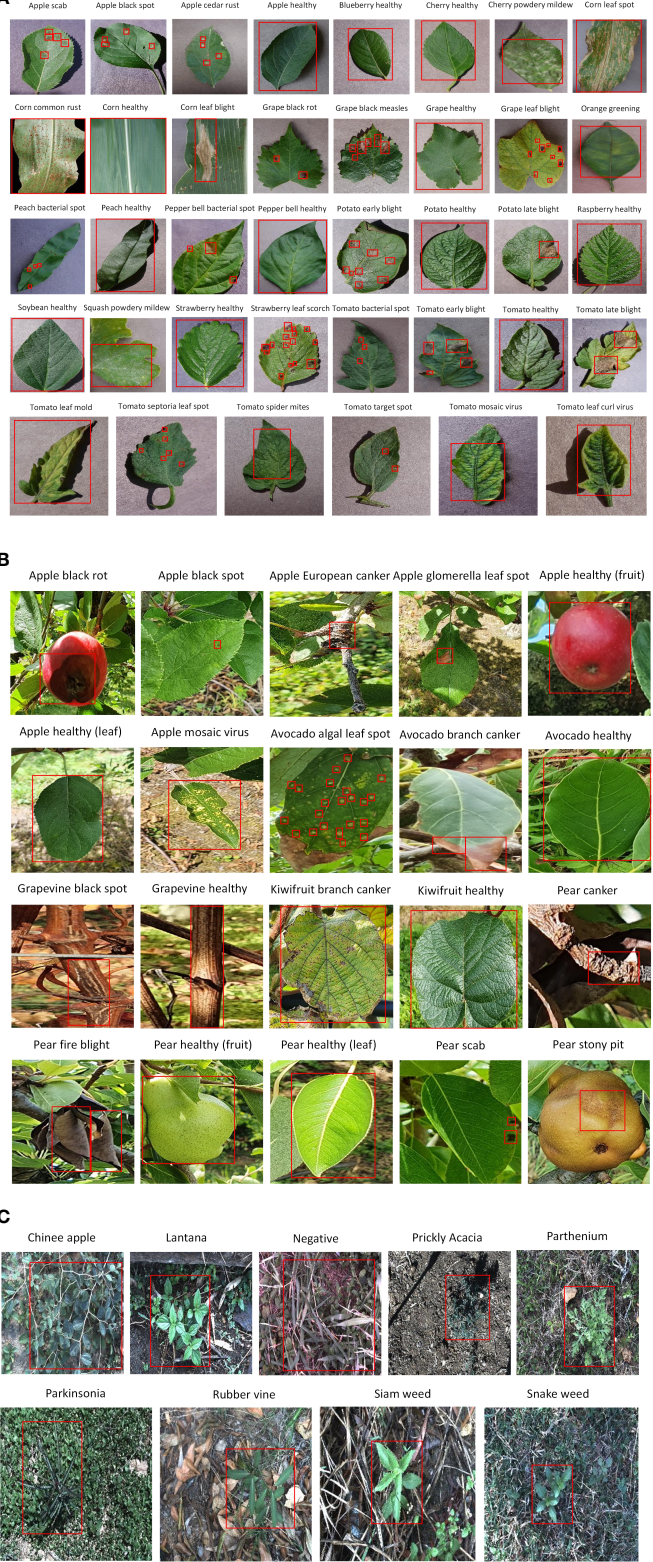
Annotated example of each class from agricultural datasets. **(A)** PlantVillage. **(B)** NZDLPlantDisease-v1. **(C)** DeepWeeds.

Three agricultural datasets were selected to extract the distinct features of different diseases in the plant species. The first dataset was the commonly used plant disease dataset, called PlantVillage (PV). The PV dataset contained 38 classes of healthy and diseased plant leaves from 14 plant species (Hughes and Salathé, 2015). The dataset images were generated in a laboratory-controlled environment. The former version of the proposed dataset series, NZDLPlantDisease-v1 (Saleem et al., 2022a), was selected. This dataset comprises 20 classes of healthy and defective plant leaves, stems, and fruits from New Zealand’s five important horticultural crops. All the dataset images were collected in a real horticultural environment. The third dataset is the DeepWeeds dataset, which contains eight classes of weeds and a negative/non-weed class from northern Australia (Olsen et al., 2019). The DeepWeeds dataset was also considered a real agricultural background when collecting the dataset images. An annotated example of each class from these agricultural datasets is presented in **Figure 4**.

### Deep learning framework and models

In this study, TensorFlow object detection APIs 1 and 2 were used to train the DL models. The pre-trained weights on the Common Object in Context (COCO) dataset was used to train the DL architectures in the first phase. To increase the training speed, an NVIDIA Graphical Processing Unit GeForce GTX 1080 Ti was used with the following specifications: 11 GB memory, 3584 CUDA cores, 484 GB/s memory bandwidth, and a 1582 MHz boost clock. To accelerate training, the CuDNN library was imported into the system.

For this research, various state-of-the-art DL models were trained, including RetinaNet (Lin et al., 2017), EfficientDet (Tan et al., 2020), faster region-based convolutional neural network (Faster RCNN) (Ren et al., 2015), single-shot multibox detector (SSD) (Liu et al., 2016), and region-based fully convolutional network (RFCN) (Dai et al., 2016). Some models were trained using different feature extractors, as available in the TensorFlow API. The performance of the DL architectures was evaluated by mean average precision (mAP), which was used to analyze the performance of several DL models, such as Faster RCNN (Ren et al., 2015), SSD (Liu et al., 2016), and RFCN (Dai et al., 2016).

### Optimization techniques

#### Image resizers and interpolators

The first step in the performance optimization of the best-obtained DL model was studying the effects of two image resizing techniques: aspect ratio (AR) and fixed-shape (FS) resizers. Image resizers were applied to the interpolators, including bilinear, area, bicubic, and nearest neighbor.

#### Weight initializers

The next step was to evaluate the three weight initialization methods. First, the truncated normal (TR) was analyzed to remove dead neurons owing to the use of the ReLU activation function. Then, scaling variance (SV) was applied, which was beneficial for maintaining the variance of the output layer with the input layers (He et al., 2015). The random normal (RN) was also tested, which helped to create tensors through normal distribution.

#### Batch normalization

The effects of batch normalization (BN) were also analyzed and used to solve the internal covariate shift. This neural network (NN) problem occurs because of the variation in the input of the NN distribution with the variation in the parameters of the previous layer (Ioffe and Szegedy, 2015). Furthermore, this method accelerates neural network training.

#### DL optimizers and hyperparameter selection

Three well-known DL optimization algorithms were used: stochastic gradient descent (SGD) with momentum (Ruder, 2016), adaptive moment estimation (Adam) (Kingma and Ba, 2014), and root mean square propagation (RMSProp) (Hinton et al., 2012). Hyperparameters were selected using the random search method (Bergstra and Bengio, 2012), such as the learning rate (LR), momentum (mom), epsilon (eps), and discounting factor (rho), as presented in **Table 2**.

**Table 2:** Hyperparameters of deep learning optimizers.

DL optimizers	DL meta-architectures	Hyperparameters
SGD with momentum	EfficientDet	LR = 3 x 10-3, mom = 0.9
	Faster RCNN ResNet-50	LR = 2 x 10-4, mom = 0.9
	Faster RCNN ResNet-101	LR = 2 x 10-4, mom = 0.9
	Faster RCNN Inception-v2	LR = 1 x 10-4, mom = 0.9
	R-FCN ResNet-101	LR = 3 x 10-4, mom = 0.9
	SSD Inception-v2	LR = 3 x 10-3, mom = 0.9
	SSD MobileNet-v2	LR = 2 x 10-4, mom = 0.9
	SSD ResNet-50 (RetinaNet)	LR = 4 x 10-4, mom = 0.9
SGD with momentum	Faster RCNN Inception ResNet-v2	LR = 3 x 10-4, mom = 0.9
Adam		LR = 1 x 10-4, eps = 1.0-2
RMSProp		LR = 2.5 x 10-4, rho = 0.9, mom = 0.9, eps = 1 x 10-3

### Validation techniques

#### External dataset

The effectiveness of this study was validated by generating an external dataset. This dataset contains the healthy and diseased plant classes of NZDLPlantDisease-v2 using a random Google search. This was done to ensure that the proposed methodology is valid in different agricultural and environmental situations.

#### Cross-validation method

This method considers the class imbalance problem (the number of samples is different for each class) in the dataset for validation of the proposed method. The stratified five-fold cross-validation technique was used to maintain the original sample size of each class in each fold (He and Ma, 2013). Otherwise, a biased distribution of the dataset images could have occurred if all the samples had been randomly mixed and divided into a certain number of folds.

## Results

This study aimed to detect plant diseases using a newly generated dataset named NZDLPlantDisease-v2. The proposed approach is divided into two phases, each containing two steps. The first step of the initial phase is dedicated to the collection, augmentation, and annotation of the dataset, as explained in the previous section. The subsequent steps are described in this section. Following the methodology shown in **Figure 1**, the results from the second step of the first phase to the second step of the final phase are presented in this section.

### Phase 1: Selection of the best DL architecture and data augmentation technique using NZDLPlantDisease-v2

#### Training through pre-trained COCO weights

Initially, the DL architectures were trained on the original images (without augmentation) of the proposed dataset. The two best DL architectures that achieved the highest mAP were selected. Further analysis of both DL models was performed by training them using different augmentation techniques. As default configurations, models such as Faster RCNN and RFCN were trained with aspect ratio image resizing methods with a minimum of 600 and a maximum of 1000 pixels, respectively. However, in EfficientDet, the same resizer with dimensions of 512 was used. Furthermore, the RetinaNet and SSD models were trained with a fixed shape resizer with 300 × 300 pixels.

The tradeoff between accuracy and training time was addressed by testing different batch sizes; the most feasible was found to be 4. SGD with momentum was the default optimizer for training each model. Using empirical observations, all DL models were trained for 200k iteration steps. However, the required number of iteration steps for the training convergence is different for each model. For example, Faster RCNN Inception ResNet-v2 required the least number of iterations, approximately 110k steps. SSD Inception-v2 required the highest number of iteration steps, approximately 190k steps. However, RFCN required 120k steps, Faster RCNN ResNet-50, Faster RCNN ResNet-101, Faster RCNN Inceptionv2 required 160k iteration steps, SSD MobileNet-v2, and RetinaNet required 170k steps, whereas EfficientDet required 180k steps. In terms of the computation/training time, SSD MobileNet was the fastest model, requiring only 5.5 hours, and Faster RCNN Inception ResNet-v2 was the slowest model, which took approximately 18 hours to complete the training. The main observations from this step are as follows.

**Figure 5:**
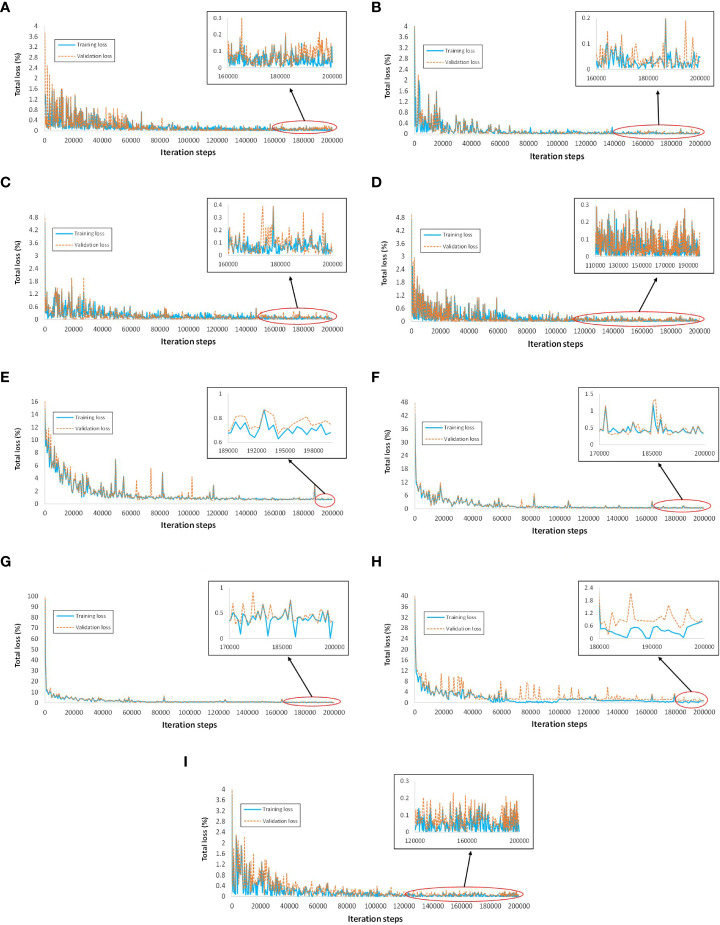
Training and validation loss plots of various DL architectures. **(A)** Faster RCNN ResNet-50. **(B)** Faster RCNN ResNet-101, **(C)** Faster RCNN Inception-v2. **(D)** Faster RCNN Inception ResNet-v2. **(E)** SSD Inception-v2. **(F)** SSD MobileNet-v2. **(G)** RetinaNet. **(H)** EfficientDet. **(I)** RFCN ResNet-101.

**Figure 6:**
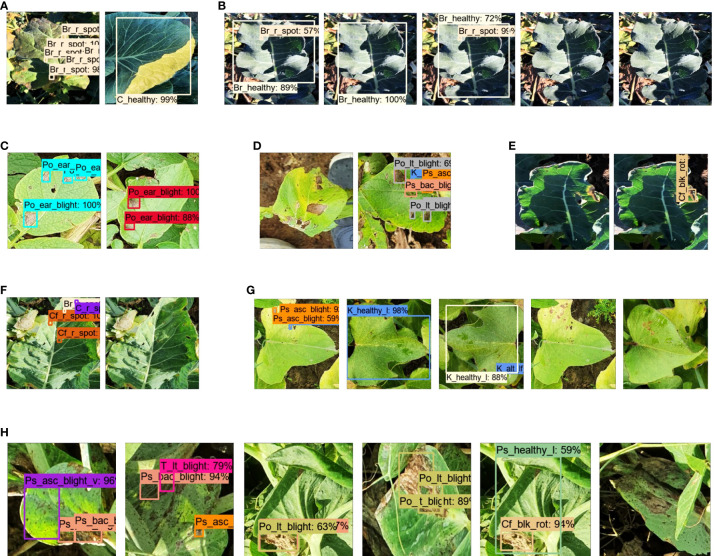
Detection results for the first step. **(A)** True positive by Faster RCNN Inception ResNet-v2 for broccoli ring spot and healthy cabbage (leaves). **(B)** False positive for broccoli ring spot by Faster RCNN ResNet-50, SSD Inception, SSD MobileNet, and the last two are undetected (false negative) by RetinaNet, EfficientDet for broccoli ring spot. **(C)** True positive by Faster RCNN Inception ResNet-v2 and ResNet-101 for potato early blight. **(D)** False negative and false positive by SSD MobileNet and EfficientDet, respectively for potato early blight. **(E)** False negative and false positive by Faster RCNN ResNet-101 and Inception ResNet-v2, respectively for broccoli black rot. **(F)** False positive by Faster RCNN ResNet-50 and false negative by SSD Inception-v2 for cauliflower ring spot. **(G)** False positive by EfficientNet, RFCN ResNet-101, Faster RCNN Inception ResNet-v2, and false negative by SSD MobileNet, RetinaNet for kumara Alternaria leaf spot. **(H)** False positive by Faster RCNN ResNet-50, ResNet-101, Inception-v2, SSD with Inception-v2, and MobileNet-v2; false negative by EfficientDet for peas bacterial blight.

- The training and validation plots of all DL models did not show any sign of overfitting, as the losses converged and had no abrupt increase in the loss after the final iteration step.
- The training and validation profiles of the DL architecture are shown in **Figure 5**. The Faster RCNN ResNet-101 had the lowest training loss of approximately 0-0.1%, followed by Faster RCNN ResNet-50 and RFCN with 0-0.15% loss, Faster RCNN Inception-v2 with 0-0.2% loss, and Faster RCNN Inception ResNet-v2 with 0-0.25% loss. The rest of the DL models attained a comparatively higher training loss between 0-0.5%, 0-0.7%, 0-0.8%, and 0.5-1% for RetinaNet, SSD Inception-v2, EfficientDet, and SSD MobileNet-v2, respectively. Similarly, the lowest validation loss was obtained by Faster RCNN ResNet-101 followed by RFCN ResNet-101, Faster RCNN ResNet-50, and Faster RCNN Inception ResNet-v2.
- In terms of mAP, Faster RCNN Inception ResNet-v2 achieved the highest value of 63.74%, followed by Faster RCNN ResNet-101 with 60.34%, as shown in **Table 3**. This was due to the high (>80%) average precision (> 80%) of 11 and 10 classes by Faster RCNN trained with Inception ResNet-v2 and ResNet-101 feature extractors, respectively. The selection of the two best DL models was validated by retraining each model using all augmentation techniques. Similar models achieved the best results; for example, Faster RCNN Inception ResNet-v2 achieved 47.83% and Faster RCNN ResNet-101 achieved 45.25% mAP.
- EfficientDet and RetinaNet achieved the lowest mAP owing to several undetected classes, as indicated by the zero AP in **Table 3**.
- It was also observed that few classes were successfully detected with Faster RCNN Inception ResNet-v2 that were undetected (false negative) or wrongly identified (false positive) with other DL models, such as broccoli ring spot (Br_r_spot) and cabbage healthy (C_healthy), as shown in (**Figures 6A, B**). Similarly, potato early blight attained the highest average precision with the best-selected DL models (**Figure 6C**). However, SSD MobileNet and EfficientDet were unable to detect some of the early blight images of potatoes (**Figure 6D**).
- Some of the classes performed well in all or most of the DL models, including bean bacterial blight, healthy cauliflower (vegetable), healthy cabbage, healthy potato stem, and healthy potato leaves.
- Although this step yielded the two best DL models, the mAP was not satisfactory. There was a significant margin in the performance improvement. This was because a few classes could not be detected, including the broccoli black rot, cauliflower ring spot, kumara Alternaria leaf spot, and pea bacterial blight, by any of the DL architectures. Several examples of the false positive and false negative results of the different DL models are presented in **Figures 6E–H**.
- After considering all data augmentation techniques, some of the classes improved their AP, such as healthy broccoli, healthy beans (leaves and vegetables), and cabbage ring spots. However, the overall performance (mAP) of the Faster RCNN Inception ResNet-v2 was degraded due to the small AP of several classes. These classes included healthy cauliflower (leaves and vegetables), kumara leaves, healthy potato early blight (leaves and stems), potato (leaves and stems), potato blight, Ascochyta blight of peas, and healthy pea leaves.

**Table 3:** Performance of deep learning meta-architectures in terms of average precision (%) of each class.

Classes of NZDLPlantDisease-v2	Deep learning architectures with feature extractors									
	Faster RCNN				SSD				EfficientDet	RFCN
	ResNet-50	ResNet-101	Inception-v2	Inception-ResNet-v2	Inception-v2		MobileNet-v2	ResNet-50 (RetinaNet)	EfficientNet	ResNet-101
Br_blk_rot	3.84	2.59	3.38	13.64	0		0	0	0	3.06
Br_healthy	29.24	36.36	18.18	36.82	45.45		43.94	38.55	23.81	21.97
Br_r_spot	23.04	41.68	33.08	72.74	6.44		12.88	0	0	34.91
B_bac_blight_v	89.61	89.61	96.16	97.49	72.73		62.63	43.10	31.49	80.42
B_healthy_l	33.33	36.36	36.82	33.33	34.55		21.97	0	0	36.36
B_healthy_v	27.27	45.45	27.27	49.91	9.09		21.97	11.49	42.10	59.69
Cf_blk_rot	55.69	59.66	49.19	58.61	22.58		20.43	8.29	53.18	61.08
Cf_healthy_l	31.19	30.5	30.83	54.7	43.01		45.45	0	0	16.41
Cf_healthy_v	90.91	90.91	81.82	90.91	81.82		90.91	59.45	65.99	90.91
Cf_r_spot	17.77	4.85	0.91	14.77	0		0	0	0	3.62
Cf_s_rot	49.57	63.64	70.99	36.36	49.55		45.45	0	11.25	60.29
C_healthy	50.69	81.21	58.56	94.54	80.56		80.68	0	10.05	69.43
C_r_spot	66.67	98.61	72.03	96.72	22.98		23.03	35.57	30.13	100
K_healthy_l	81.82	80.17	90.08	81.82	56.01		80.91	42.98	27.65	81.82
K_alt_lf_spot	9.09	23.48	0	37.02	0		0	0	0	28.83
Po_lt_blight_s	57.52	36.01	28.44	32.55	34.22		32.83	0	0	51.27
Po_ear_blight	71.08	86.48	72.44	88.45	59.87		54.17	60.02	43.66	67.87
Po_ear_blight_s	42.17	59	67.65	69.19	24.84		23.97	21.33	28.18	57.85
Po_healthy_l	88.76	86.56	90.91	78.1	70.69		79.44	19.74	38.59	96.28
Po_healthy_s	90.08	90.91	80.17	89.39	72.73		72.73	77.73	53.88	88.16
Po_lt_blight	36.58	51.23	57.56	52.99	0		4.55	0	0	44.21
Ps_asc_blight	72.24	79.98	78.97	71.43	54.21		35.86	0	10.25	79.67
Ps_asc_blight_v	89.26	79.17	87	87.63	100		72.73	46.52	4.83	81.82
Ps_bac_blight	18.08	31.47	26.71	14.16	4.55		6.93	0	0	41
Ps_healthy_l	100	98.6	97.78	90.91	100		89.17	63.38	74.12	89.4
Ps_healthy_v	90.91	81.82	90.91	90.91	90.91		79.9	0	25.88	81.82
T_healthy_l	72.73	72.73	81.82	81.82	72.73		81.82	37.81	35.91	72.73
T_lt_blight	42.93	50.44	37.13	67.74	35.45		27.27	0	0	63.49
mAP (%)	54.72	60.34	55.96	63.74	44.46		43.27	20.21	21.82	59.44

#### Category-wise study of data augmentation techniques

To understand the effects of the data augmentation techniques on the DL model, we divided them into five categories, as explained in the previous section. The two best DL architectures were retrained for each category. The results are discussed below.

**Figure 7:**
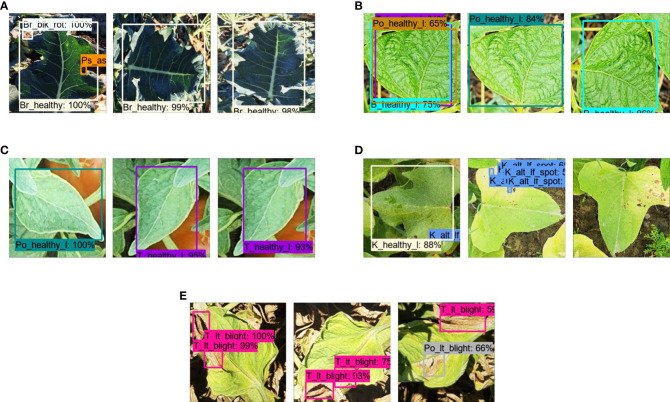
Plant disease detection by different augmentation techniques. **(A)** False positive by OC and true positive by OT and OTCN. **(B)** False positive by OO, OC, and true positive by OT for healthy beans (leaves). **(C)** False positive by OC and true positive by OT and OCN for healthy tomato leaves. **(D)** False positive by OO, true positive by OT, and false negative by OTCN for kumara Alternaria leaf spot. **(E)** True positive by OT and OCN, and false positive by OC for tomato late blight.

- Among the five augmentation categories, OT achieved the best results in terms of the highest mean average precision. The performance of the Faster RCNN Inception ResNet-v2 model was significantly improved as compared to the non-augmented categories, with a difference of 18.34% in mAP.
- Nine classes were prominently detected after the application of the OT category. These classes included healthy broccoli, healthy beans, healthy cauliflower black rot, healthy cauliflower (leaves), soft cauliflower rot, kumara Alternaria leaf spot, potato late blight (stem) potato early blight (stem), and Ascochyta blight, which improved the individual AP by 63.18%, 66.26%, 17.15%, 43.04%, 63.18%, 61.91%, 48.38%, 25.59, and 27.55%, respectively.
- The OT category also attained the best performance for Faster RCNN ResNet-101. The mAP improved by approximately 10.49% compared to the results obtained with original/non-augmented images.
- It was further noticed that OTCN and OO achieved the lowest mAP for both DL models, followed by OC and OCN categories.
- The change in color/noise inclusion affected the detection performance, as shown in **Figure 7A**. When the image of a healthy broccoli leaf was tested, the background element was wrongly identified as broccoli black rot disease, and the blackness in the leaf was recognized as pea Ascochyta blight. Some other examples of true positive and false positive results with the respective augmentation categories are presented in **Figures 7B–E**.
- Although this step of the study improved the performance of the Faster RCNN Inception ResNet-v2, few classes achieved a low AP <50%, such as broccoli black rot, healthy beans (vegetables), cauliflower ring spot, potato late blight, and peas bacterial blight.

### Phase 2: Performance optimization by weights obtained from agricultural datasets

This phase of the research was divided into two steps: obtaining the optimized weights on agricultural datasets using different techniques and using the knowledge/features extracted from the agricultural datasets to the proposed dataset.

#### Effects of image resizers, interpolators, weight initializers, batch normalization, and DL optimizers on agricultural datasets

First, the best-obtained DL model, Faster RCNN Inception ResNet-v2, was trained on each agricultural dataset using default settings. Subsequently, various image resizers, interpolators, weight initializers, batch normalization, and DL optimizers are applied to determine the most suitable combination. The weights obtained through the best-performing configuration in terms of the highest mAP and highest number of detected classes (for each dataset) were saved for reuse in the later stage of this phase of the research. A summary of the results by default and optimized settings is presented in **Table 4**.

**Table 4:** Summary of each step of the proposed methodology.

Steps of theproposed approach		Training Specifications (Image resizers, interpolators, initializer, batch normalization, and DL optimizers)	Training and testing performance					Analysis and remarks
			DL models	Training time (h)	Training loss (%)	Validation loss (%)	mAP (%)	
Obtain the best-suited DL architecture		AR with bilinear TN, SV No BN SGD	Faster RCNN Inception ResNet-v2	18	0.13	0.35	63.74	Two of the best DL models achieved the highest mAP; some of the classes were not detected.
			Faster RCNN ResNet-101	12	0.06	0.2	60.34	
Application of data augmentation techniques		Original and translation/rotational changes Default training settings	Faster RCNN Inception ResNet-v2	18	0.425	0.61	82.08	Both DL architectures achieved better results after adding translational/rotational images to the training dataset.
			Faster RCNN ResNet-101	12	0.375	0.52	70.83	
Training on agricultural datasets	PV	Default settings	Faster RCNN Inception ResNet-v2	18	0.54	0.75	61.11	The Faster RCNN Inception ResNet-v2 was trained on different datasets with default settings.
	NZDL-1		Faster RCNN Inception ResNet-v2	18	0.42	0.62	71.48	
	DW		Faster RCNN Inception ResNet-v2	18	0.63	0.75	80.81	
Weight optimization on agricultural datasets	PV	FS with bilinear, RN, without BN, SGD	Faster RCNN Inception ResNet-v2	12	0.46	0.64	69.23	Different performance enhancement techniques were applied to the agricultural datasets to obtain optimized weights; the best combination of image resizer/interpolator, initializer, batch normalization, and DL optimizers was found to depend on the selected datasets.
	NZDL-1	FS with bicubic, RN, BN, SGD	Faster RCNN Inception ResNet-v2	12	0.31	0.46	73.32	
	DW	FS with bilinear, TR, SV, without BN, RMSProp	Faster RCNN Inception ResNet-v2	12	0.49	0.66	84.36	
Retrain through the best-optimized weights on NZDLPlantDisease-v2		PV dataset: FS with bilinear, RN, without BN, SGD	Faster RCNN Inception ResNet-v2	12	0.34	0.50	91.33	Used optimized weights to train Faster RCNN Inception ResNet-v2 on NZDLPlantDisease-v2 dataset; achieved a significantly higher mAP.
Compare the results with optimized weights on the COCO dataset		FS with bilinear, TR, SV, without BN, SGD	Faster RCNN Inception ResNet-v2	12	0.39	0.56	85.85	Another comparison was made with the optimized weights on the COCO dataset.
Validation of the approach		External dataset	–	–	–		93.20	Most of the classes attained a high mAP with the optimized weights.
		Stratified k-fold cross-validation	–				88.53	No noticeable differences in the performance of the final model were found; few classes can be considered in future studies.

##### Optimization on the PlantVillage dataset

After training the Faster RCNN Inception ResNet-v2 with default settings, the effects of the aspect ratio and fixed shape resizer were studied on the PlantVillage dataset. Image resizers were evaluated along with interpolators to obtain the best-suited arrangement. In this regard, an aspect ratio resizer with a bilinear interpolator achieved an mAP of 61.11%. It was improved by 3.17% by training through a fixed-shape resizer trained with a bilinear interpolator with a pixel size of 300 × 300 pixels. The reason for this was the true positive detection results in the classes including apple scab, healthy peach, healthy soybean, and squash powdery mildew. Furthermore, the model performed well after adding pixels by taking the average of the color values of the neighboring pixels by bilinear interpolation.

Similarly, a performance enhancement of 4.95% in the mAP was attained after the application of a random normal initializer. Therefore, the Faster RCNN model initialized with tensors having a normal distribution was found to be the best method for initializing the weights of the neural network. Next, batch normalization was applied with different values of epsilon and decay. It was empirically found that a 0.9 value for decay and an epsilon of 0.01 were the most suitable values. However, the mAP did not improve and was 65.68%. Therefore, the next step was applied in the absence of batch normalization. The last step was to study the effects of DL optimizers on the Faster RCNN model for the PlantVillage (PV) dataset. The Adam optimizer could not extract the features of several classes and achieved a low mAP of 26.53%. However, RMSProp achieved an mAP of 61.695%, which was not an improvement over the default (SGD with momentum) optimizer. Therefore, the final settings for the PV dataset were the use of a fixed-shape resizer with a bilinear interpolator, random normal initializer, SGD with momentum optimizer, and no batch normalization, as presented in **Table 4**.

##### Optimization on the NZDLPlantDisease-v1 dataset

Like the PV dataset, the weights of the Faster RCNN model were optimized using the NZDLPlantDisease-v1 dataset with the same framework/steps. The default configuration reached 71.48% mAP. However, an improvement of 0.28% in the mAP was observed when the input images were resized using the fixed shape method with a bicubic interpolator. A few classes, such as apple mosaic virus and healthy pear (leaves), achieved 17.01% and 50.49%, respectively, higher AP with fixed shape (bicubic) compared to aspect ratio (bilinear). However, only one class (pear scab) achieved better output with the default resizer/interpolator combination. Faster RCNN Inception ResNet-v2 also performed well for the NZDLPlantDisease-v1 dataset with a random normal initializer, and an enhancement of 1.1% mAP was observed. There was a small difference in the AP of the healthy/disease classes compared with the default initializer. Batch normalization with epsilon and decay was applied at 0.5 and 0.01, respectively. The mAP improved from 72.86% to 73.32%. Again, SGD with momentum performed well compared to the other two DL optimization algorithms.

##### Optimization on the DeepWeeds dataset

The last selected dataset is related to other agricultural application (weed identification). It also started with an evaluation using the default settings. Like the other two datasets, the fixed-shape resizer worked well for this dataset. The bilinear interpolation method achieved the highest mAP among all interpolators and achieved 84.20% mAP compared to 80.81% with the default resizer. However, in contrast to the previous datasets, DeepWeeds performed better on truncated normal and scaling variance initializers than on random normal initializers. Thus, it can be said that a truncated normal distribution and adaptation of the scale to the shape of weights resulted in a better initialization for weed detection. However, batch normalization did not work for this dataset, and a small performance degradation was observed, with a difference of almost 0.3%. When evaluating the DL optimizers, RMSProp attained the best outcomes and slightly improved the mAP to 84.36%.

##### Transfer learning by optimized weights

**Figure 8:**
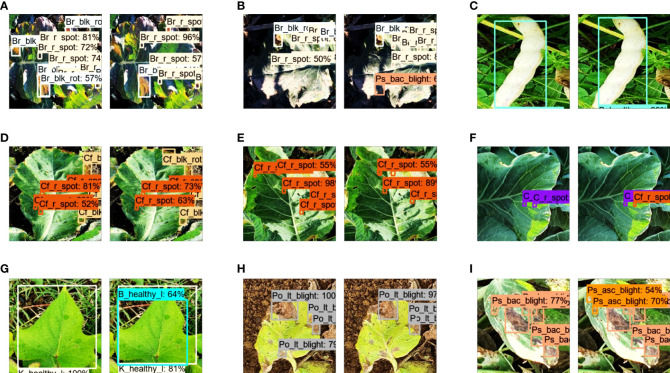
Comparison of various healthy and disease classes by optimized final model and results obtained in the previous step. **(A)** Broccoli black rot. **(B)** Broccoli ring spot. **(C)** Bean healthy vegetable. **(D)** Cauliflower black rot. **(E)** Cauliflower ring spot. **(F)** Cabbage ring spot. **(G)** Kumara healthy. **(H)** Potato late blight. **(I)** Peas bacterial blight.

The last step of this research evaluates the Faster RCNN Inception ResNet-v2 model on the proposed dataset using optimized weights from each selected agricultural dataset. In this regard, the training and testing performances are analyzed, and the effects of updated/new weights are studied. The significance of this step is presented by comparing the results obtained in the previous phase (step 2) and optimizing the weights using a large dataset (COCO). Some important observations from this research are presented below:

- The mAP of the Faster RCNN model was increased to 91.33% by optimizing the weights through the PV dataset and improved by a margin of 9.25% in mAP. The total training loss was lower than 0.34% owing to a considerable reduction of 0.12 and 0.16% in region proposal network (RPN) localization and box classifier classification losses, respectively. Moreover, a small reduction in box classifier localization and RPN objectness loss was observed at 0.05 and 0.01%, respectively. Furthermore, the validation loss was reduced to 0.5%. Almost all nine classes improved their detection outcomes and attained > 80% AP. These classes include broccoli black rot, broccoli ring spot, bean healthy (vegetable), cauliflower black rot, cauliflower ring spot, cabbage ring spot, kumara healthy, potato late blight (leaves), and pea bacterial blight. An example of each class is shown in **Figure 8**.
- In **Table 4**, the mAP with optimized weights by NZDLPlantDisease-v1 (former version in the series of the proposed plant disease dataset) was 87.68% and therefore increased by 5.6% compared to the previous step. There are several reasons for this improvement, as observed by the training and testing profiles of the model. First, the total training loss was reduced to approximately 0.36%, which was 0.425% when the model was trained using COCO weights. This reduction in the total loss was due to a small decrease in RPN objectness and localization loss of 0.01% and 0.055%, respectively. These losses produce a substantial improvement in the mAP. However, no noticeable changes were observed in the box classifier losses. Second, several classes were detected, or their individual AP was improved, such as broccoli ring spot, cauliflower black rot, cabbage ring spot, healthy kumara, and pea bacterial blight, by 30.58%, 23.34%, 23.75%, 28.94%, and 24.56%, respectively.
- However, the performance of the Faster RCNN model was not improved by using the weights obtained on the DeepWeeds dataset. The mAP was 80.73% with performance degradation in the classes such as broccoli healthy, cauliflower healthy (leaf), and potato early blight (stem).
- Finally, a similar weight optimization framework was applied to the new dataset using pre-trained weights on a large dataset (COCO). The best configurations were a fixed image resizer with a bilinear interpolator, truncated normal and scaling variance initializers without batch normalization, and an SGD optimizer. It was found that the Faster RCNN Inception ResNet-v2 attained 85.85% mAP, which was improved by 3.77% compared to the previous phase, but 5.48% and 1.83% lower than the mAP obtained through the weights of the PV dataset and NZDLPlantDisease-v1, respectively.

## Discussion

In this section, a comprehensive analysis is presented to explain the substantial improvement in the mAP using the proposed methodology for the detection of plant disease. Finally, the effectiveness of the approach is highlighted using two methods, along with describing some limitations of the study.

### Analysis of the results obtained in the first and second phases of the research

- The first phase of the research was dedicated to finding the best-suitable DL model trained on the proposed dataset using pre-trained weights on a large COCO dataset along with the best data augmentation method. The main finding of this step was that the Faster RCNN Inception ResNet-v2 achieved the best results in terms of the highest mean average precision in the absence and presence of augmented images. However, some classes achieved a low AP. Interestingly, several classes performed worse when the model was trained using all augmentation (OTCN) techniques. Therefore, different data augmentation categories were investigated before attempting any improvement in the performance of the DL model.
- The Faster RCNN model attained the best results when it was trained with the translational/rotational (OT) data augmentation method. There could be several reasons for obtaining unsatisfactory results in different categories, except for the OT. First, the number of training images for the OO category was insufficient to extract the distinct features of the healthy and disease classes. After a change in the brightness, contrast, and sharpness of the images, the symptoms of the plant disease might become similar to other healthy/diseased classes of the same plant species or another, resulting in false positive outcomes. Furthermore, the features of plant diseases resemble the background elements after the change in color or the inclusion of noise in the input images as shown in **Figure 7A** (Barbedo, 2018). Therefore, the Faster RCNN model might not be able to extract specific features or symptoms of plant diseases.
- Furthermore, OT augmentation has practical importance. This is because the location/position of the disease spot and plant organs are different in real agricultural fields. Therefore, if the DL model performs well on translated/rotated images, it can be used in a complex agricultural environment.
- The second phase aimed to study the effects of several performance optimization methods on the agricultural datasets to obtain the optimized weights of the Faster RCNN model. Finally, the optimized weights from these agricultural datasets were tested on the proposed dataset.
- It can be observed from **Table 4** that each of the selected datasets required different specifications. The combination of various techniques depended on the dataset because all three datasets had several fundamental dissimilarities, for example, background environment, number of dataset images, number of classes, and image quality. In addition, the optimized weights produced a significant improvement in the performance of Faster RCNN Inception ResNet-v2 for the three datasets. This demonstrates the importance of the performance optimization pipeline (Saleem et al., 2022b).
- From the results, it can be observed that the performance of the Faster RCNN model was improved using weights optimized by other plant disease datasets. The weights from the weed datasets did not generate any significant difference in the performance of the model.
- There are several ways to understand the reasons for obtaining an ample improvement in the Faster RCNN performance by weight obtained through the PV dataset. First, PV has a greater variety of plant disease types. Therefore, various symptoms/spots were extracted for transfer learning. Next, the PV dataset had a greater number of total samples that helped learn the distinct features successfully. Hence, the useful information through the PV dataset was more suitable as compared to the pre-trained weights of the Faster RCNN by NZDLPlantDisease-v1.
- The problems highlighted in the Introduction have been addressed. This was because the final model successfully detected all the disease classes in different plant organs of various vegetables with multiple plant diseases.
- On top of that, similar performance optimization methods were applied to the proposed dataset using the pre-trained weights of the COCO/large general-purpose dataset to get the optimized weights. Then, the Faster RCNN Inception ResNet-v2 model was retrained on the proposed dataset using the new/updated weights. The results show that leveraging the knowledge learned from the agricultural dataset performed better than leveraging from the large general-purpose dataset.

### Validation of the research

The performance of the final model was validated using two methods. First, a stratified cross-validation method was used because of the class-imbalance problem. This method allows for an unbiased distribution of class samples among all folds of a dataset. The testing dataset was folded five times. The first fold was assumed to be the default testing dataset, which attained 91.33%, as explained in the previous section. The mAPs obtained from fold2, fold3, fold4, and fold5 were 91.57, 90.82, 91.14, and 91.17%, respectively. Therefore, the mAP varied with a small difference of 0.16 to 0.51%.

**Figure 9:**
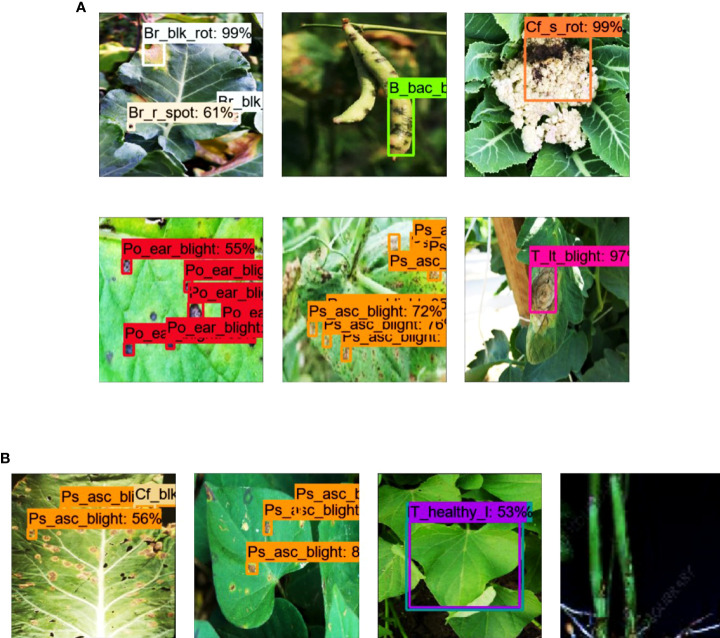
Results with the external testing dataset. **(A)** True positive outcomes. **(B)** False positive and false negative results.

Subsequently, another validation was performed by testing the final model on an external dataset (generated through a random Google search). Overall, 14 classes achieved an AP of more than 90%, including broccoli black rot, broccoli healthy, bean bacterial blight, cauliflower black rot, cauliflower healthy (leaves and vegetables), potato early blight (leaves and stems), potato late blight (stem), peas Ascochyta blight (leaves and vegetables), peas healthy (vegetables), tomato healthy, and tomato late blight. However, some classes achieved an AP of less than 83%, such as cauliflower ring spot, kumara Alternaria leaf spot, kumara healthy, and potato healthy (stem). These classes should be further studied in future research. **Figure 9** presents a few samples of the true and false positive outcomes from the external dataset.

### Limitations of the study

The performance of the Faster RCNN model was more significantly improved by using the weights optimized through PV dataset compared to NZDLPlantDisease-v1 dataset. Therefore, it is concluded that a dataset that contains a higher number of plant disease classes can contribute to improving outcomes more effectively. The proposed hypotheses could be further explored in future studies by considering at least one more plant disease dataset in both real and laboratory environments. In this way, it can also be established that the environment of the dataset (from where optimized weights are obtained) also affects the performance of the DL model.

The NZDLPlantDisease-v2 dataset contains healthy/disease classes for various organs of plant species, including beans, peas, potatoes, and cauliflower. However, some crops only contain the disease in their leaves, such as broccoli, cabbage, kumara, and tomato. Therefore, data collection should be continued with other plant organs of these vegetables. Moreover, due to time constraints and weather conditions, a few other important New Zealand vegetables, such as onion, squash, and sweet corn, could not be considered.

The problem of multiclass plant diseases in a single organ at a time has been addressed for only four classes. Hence, this problem has been analyzed in a limited manner. Moreover, the data annotation method is a lengthy process for a large number of classes. The more difficult part is to annotate the images with the multiclass problem, as each image must be labeled multiple times (for each disease). Therefore, if a plant organ suffers from a greater number of diseases, annotation time increases significantly which can lead to reducing the accuracy of data annotation. Hence, DL-based research still requires human effort before training/evaluating models to perform delicate tasks such as plant disease detection.

## Conclusions and future recommendations

This research has addressed an important agricultural problem of identifying plant diseases in New Zealand vegetables by deep learning. In this regard, a new dataset named NZDLPlantDisease-v2 was generated, which contains 28 healthy/disease classes in eight plant species. This dataset also contains complex agricultural challenges, including multiple diseases in a single vegetable plant organ, diseases in different plant organs, and variations in real agricultural environments.

A two-step transfer learning approach based on weight optimization using different techniques on agricultural datasets was proposed. In the first phase, the best-suited DL architecture was found to be Faster RCNN Inception ResNet-v2 which attained the highest mAP using pre-trained weights on the COCO dataset. Then, the Faster RCNN model with translated/rotated data augmentation techniques improved the performance of the DL model, with an mAP of 82.08%. In the second phase, the knowledge/weights extracted from the agricultural dataset were transferred to learn the features of the classes of the proposed dataset. In this regard, three agricultural datasets were considered: two plant disease datasets in a controlled and real environment and a weed dataset in complex agricultural conditions. The weights obtained using the PlantVillage dataset significantly improved the performance of the Faster RCNN model. The optimization of weights was obtained having the best configurations consisting of a fixed-shape resizer along with a bilinear interpolator, random normal initializer, without batch normalization, and the SGD optimizer. The final mAP was 91.33%, and each class attained more than 80% AP. The optimized DL model outperformed the results obtained in the first step and the optimized weights using a general-purpose dataset (COCO). The effectiveness of the proposed approach was validated using a stratified k-fold cross-validation method and external testing dataset.

Overall, this research has demonstrated an extended strength of deep transfer learning for plant disease detection. The proposed methodology can be used in other agricultural applications. To extend this work, various concepts can be explored to visualize the feature extraction of healthy/diseased plant organs, such as t-distributed stochastic neighbor embedding (t-SNE) plots. Moreover, a modified DL model should be proposed to reduce the training/computation time. Furthermore, a similar approach can be tested for segmentation tasks using the latest DL architectures, such as DeepLab-v3, U-Net, feature pyramid network (FPN), and pyramid scene parsing network (PSPNet). A more in-depth analysis can be performed using other performance metrics (Wu and Zhou, 2017) for the multiclass plant disease detection problem.

## Data availability statement

The dataset presented in this study is made publicly available in a GitHub repository: https://github.com/kmarif/NZDLPlantDisease-v2.

## Author contributions

MHS and KMA designed the research and proposed the methodology. MHS curated the dataset, performed the experiments, and wrote the original draft. KMA reviewed and edited the manuscript. JP and KMA obtained funding and supervised this research. All authors contributed to the article and approved the submitted version.

## Funding

This research was funded by the Ministry of Business, Innovation, and Employment (MBIE), New Zealand, Science for Technological Innovation (SfTI) National Science Challenge.

## Acknowledgments

The authors are grateful to Dr. Carl Mesarich (School of Agriculture and Environment, Massey University) for providing the common and scientific names for plant diseases. The authors also thank Fakhia Hammad and Muhammad Taha for their help with the annotation of the dataset images.

## Conflict of interest

The authors declare that the research was conducted in the absence of any commercial or financial relationships that could be construed as a potential conflict of interest.

## Publisher’s note

All claims expressed in this article are solely those of the authors and do not necessarily represent those of their affiliated organizations, or those of the publisher, the editors and the reviewers. Any product that may be evaluated in this article, or claim that may be made by its manufacturer, is not guaranteed or endorsed by the publisher.
